# Pyrotinib combined with CDK4/6 inhibitor in HER2‐positive metastatic gastric cancer: A promising strategy from AVATAR mouse to patients

**DOI:** 10.1002/ctm2.148

**Published:** 2020-08-13

**Authors:** Zuhua Chen, Yingying Xu, Jifang Gong, Furong Kou, Mengqi Zhang, Tiantian Tian, Xiaotian Zhang, Cheng Zhang, Jian Li, Zhongwu Li, Yumei Lai, Jianjun Zou, Xiaoyu Zhu, Jing Gao, Lin Shen

**Affiliations:** ^1^ Key laboratory of Carcinogenesis and Translational Research (Ministry of Education/Beijing), Department of Gastrointestinal Oncology Peking University Cancer Hospital and Institute Beijing China; ^2^ Department of Oncology, Tongji Hospital, Tongji Medical College Huazhong University of Science and Technology Wuhan China; ^3^ Key laboratory of Carcinogenesis and Translational Research (Ministry of Education/Beijing), Department of Pathology Peking University Cancer Hospital and Institute Beijing China; ^4^ Jiangsu Hengrui Medicine Co, Ltd Jiangsu China; ^5^ National Cancer Center/National Clinical Research Center for Cancer/Cancer Hospital & Shenzhen Hospital Chinese Academy of Medical Sciences and Peking Union Medical College Shenzhen China

**Keywords:** CDK inhibitor, pyrotinib, refractory mechanisms

## Abstract

**Background:**

Pyrotinib was well tolerated but its efficacy was unsatisfactory in patients with HER2‐positive gastric cancer (GC) (NCT02378389). This study was to optimize the efficacy of pyrotinib.

**Methods:**

Human GC cell lines and AVATAR mice were used to explore the refractory mechanisms of pyrotinib. A pyrotinib‐combined strategy was proposed, which was validated in preclinical AVATAR mouse and in clinical patients enrolled in a phase I clinical trial (NCT03480256).

**Results:**

Dysregulation of CCND1‐CDK4/6‐Rb axis might be the key to pyrotinib refractory. The strategy of pyrotinib combined with a CDK4/6 inhibitor SHR6390 was proposed and validated in preclinical AVATAR mouse, which was successfully verified in clinical patients. For five patients treated with pyrotinib plus SHR6390 who had available response evaluation, the best response was partial response in three patients, stable disease in one patient, and progressive disease in one patient. The progression‐free survival times were 120, 200, 532, 109, and 57 days, respectively.

**Conclusions:**

This translational study suggests that pyrotinib combined with SHR6390 may serve as a promising strategy for patients with HER2‐positive GC.

**Trial registration:**

The ClinicalTrials.gov identifiers are NCT02378389 (https://clinicaltrials.gov/ct2/show/study/NCT02378389, registered in 11 February 2015) and NCT03480256 (https://clinicaltrials.gov/ct2/show/study/NCT03480256, registered in 8 March 2018).

## 1 INTRODUCTION

Human epidermal growth factor receptor 2 (HER2) is overexpressed in approximately 10‐15% of patients with gastric cancer (GC).^1^, ^2^ Trastuzumab, a monoclonal antibody against HER2, is the only approved targeted agent that has been shown to confer overall survival benefit in the first‐line treatment of HER2‐positive advanced GC (AGC) patients.^3^ In the past decades, several clinical trials have been conducted to evaluate the efficacy of other targeting HER2 agents including pertuzumab,^4^ trastuzumab emtansine,^5^ and lapatinib^6^, ^7^ in HER2‐positive GC patients, but none of them resulted in a significant survival benefit. In addition, most of HER2‐positive AGC patients who receive trastuzumab treatment will eventually experience disease progression due to drug resistance. The strategy has remained undefined after failure of first‐line treatments in clinical practice for HER2‐positive AGC patients. At present, the development of more effective HER2‐targeting drug is still a major focus of clinical research in GC.

Pyrotinib is a novel small‐molecule tyrosine kinase inhibitor that irreversibly blocks EGFR, HER2, and HER4. Due to its good tolerability and high overall response rate, 400 mg/day pyrotinib has been approved in China for the treatment of HER2‐positive advanced or metastatic breast cancer (BC) patients previously treated with anthracycline or taxane.^8^ Meanwhile, a phase I study was conducted in our department to evaluate the safety and tolerability of pyrotinib in patients with HER2‐positive AGC (NCT02378389). The results showed that pyrotinib was well tolerated, but the antitumor activity of pyrotinib was unsatisfactory with the response rate of 21% (unpublished data described in detail in Tables S1‐S3).

This translational study was designed to investigate the potential resistant mechanisms of pyrotinib in GC using patient‐derived xenograft models named as AVATAR mouse. Subsequently, the strategy of pyrotinib combined with a CDK4/6 inhibitor SHR6390 was proposed and validated in our AVATAR mouse. Finally, a phase I clinical trial (NCT03480256) was designed to validate the safety and efficacy of pyrotinib combined with SHR6390 in patients with HER2‐positive AGC after failure of systemic treatment.

## 2 MATERIALS AND METHODS

### 2.1 Reagents and antibodies

Pyrotinib (purity ≥ 99.9%) and SHR6390 (purity ≥ 99.9%) were provided by Hengrui Medicine Co, Ltd (Jiangsu, China). Lapatinib was purchased from MedChem Express (Monmouth Junction, NJ, USA). Antibodies were purchased from Cell Signaling Technology (Boston, MA, USA) and Sigma‐Aldrich (St. Louis, MO, USA).

### 2.2 Cell culture and cell viability assay

Two HER2‐positive GC cell lines (NCI‐N87 and SNU‐216) and three HER2‐negative GC cell lines (NUGC‐4, MKN45, and HGC‐27) were purchased from the cell bank of Peking Union Medical College (Beijing, China) and confirmed by short‐tandem repeat analysis. Cells were cultured in RPMI 1640 medium (Gibco, MD, USA) supplemented with 10% fetal bovine serum (Gibco) in a humidified incubator (37°C) with 5% CO_2_. Cell viability was determined using a Cell Counting Kit‐8 (Dojindo, Kumamoto, Japan). The absorbance was measured at 450 nm using a spectrophotometer. All of the experiments were repeated at least three times.

### 2.3 The antitumor activity of pyrotinib in AVATAR mouse

We previously reported the establishment and characterization of AVATAR models using fresh gastroscopic biopsies obtained from AGC patients.^9^, ^10^ Briefly, tumor tissue with 2 × 2 × 2 mm^3^ was inoculated subcutaneously into flank of a 6‐week‐old nonobese diabetic/severe combined immunodeficiency (NOD/SCID) mouse. When the tumor volume of the AVATAR mouse reached 750 mm^3^, the tumor tissue was separated and sliced into small fragments, then re‐inoculated to other NOD/SCID mice. Mice with tumors of 150‐250 mm^3^ were randomly assigned to pyrotinib group (n = 5, pyrotinib 40 mg/kg, daily by oral gavage) and vehicle group (n = 5, physiological saline, daily by oral gavage). The tumor size and body weight were measured twice a week. The tumor volume was calculated as (Length × Width^2^) / 2. The AVATAR mouse was sacrificed after the administration cycle (21 days) or when the tumor volume reached 2000 mm^3^. Tumor growth inhibition (TGI) was determined as [1 − Δ*T* / Δ*C*] × 100% (Δ*T* and Δ*C* presented changes in tumor volume of the treatment group and control group over the course of the treatment, respectively).

### 2.4 Establishment of pyrotinib‐refractory AVATAR model

One HER2‐positive AVATAR (numbered as case 019) model, which was confirmed to be sensitive to pyrotinib, was administered continuous pyrotinib (40 mg/kg, daily by oral gavage) until the tumor was no longer sensitive to pyrotinib. Based on their responses to pyrotinib, the parental (before pyrotinib treatment), sensitive (sensitive under pyrotinib exposure), and refractory (refractory under pyrotinib exposure) models were named as 019P, 019S, and 019R, respectively.

### 2.5 Western blotting analysis

Total protein was extracted from cells and tumor tissues and western blotting was conducted as previously reported.^10^ Protein was visualized using ECL‐plus Western Blotting Detection Reagents (GE Healthcare Life Sciences, Chalfont, UK). Protein bands were quantified and normalized with Image J software.

### 2.6 Hematoxylin and eosin and immunohistochemistry staining

Tumor tissues were isolated and formalin‐fixed paraffin‐embedded tissue blocks were prepared.^10^ Hematoxylin and eosin (H&E) and immunohistochemistry (IHC) staining were conducted as previously reported and interpreted by two independent pathologists. IHC scores were interpreted as follows: 0, no staining; 1+, weak or focal staining; 2+, moderate staining; and 3+, strong staining.

### 2.7 Genomic DNA and total RNA extraction

Genomic DNA was extracted from cells and tumor tissues using the QIAamp DNA Mini Kit (Qiagen, Hilden, Germany). The total mRNA of tumor tissues was extracted using TRIzol reagent (Invitrogen). The concentrations of genomic DNA and mRNA were quantified by a Nanodrop 2000 Spectrophotometer (Thermo, Santa Clara, CA, USA).

### 2.8 Next‐generation DNA sequencing and transcriptomic sequencing

Next‐generation DNA sequencing and transcriptomic sequencing were performed and analyzed by Novogene Bioinformatics Institute (Beijing, China) as previously reported.^11^ The correlation coefficient was calculated by the fragments per kilobase million to compare the differences among samples.

### 2.9 TaqMan copy number assays

Genomic DNA was subjected to *HER2* and *EGFR* copy number analysis using TaqMan Copy Number Assays (Thermo). *RNase P* was used as the control gene. Copy number was then calculated by CopyCaller Software v 1.0 (Thermo) using the comparative *Ct* (ΔΔ*Ct*) method. Normal human control DNA was used as the reference.

### 2.10 Quantitative real‐time polymerase chain reaction

Real‐time polymerase chain reaction (RT‐PCR) was performed using SYBR Green PCR Master Mix (ABI, Carlsbad, CA, USA), with glyceraldehyde 3‐phosphate dehydrogenase (GAPDH) serving as an endogenous control. Gene‐specific primers for *CCND1*, *CDK4*, *CDK6*, and *GAPDH* are listed in Table S4.

### 2.11 Clinical trial design in AGC patients

Based on the preclinical results, a prospective phase I, single‐arm, open‐label, dose‐escalating study (NCT03480256) was designed to evaluate the safety and efficacy of pyrotinib combined with SHR6390 in patients with HER2‐positive AGC after failure of systematic treatments. Each treatment cycle was 28 days. A cohort of three oral doses was designed: (a) SHR6390 100 mg/day combined with pyrotinib 400 mg/day; (b) SHR6390 100 mg/day combined with pyrotinib 320 mg/day; (c) SHR6390 75 mg/day combined with pyrotinib 400 mg/day. SHR6390 and pyrotinib were used for 21 days and 28 days, respectively. The incidence and severity of adverse events were evaluated according to the National Cancer Institute Common Toxicity Criteria (version 4.0). Pyrotinib and SHR6390 were continued until disease progression or intolerable toxicity.

The primary endpoint was the maximum tolerated dose (MTD). The dose‐limiting toxicity (DLT) is defined by the occurrence of following drug‐related adverse reactions during the first cycle: (a) diarrhea not improved to grade 2 or less within 1 week after the best supportive care; (b) grade 3 or 4 nonhematological toxicity (except for nausea, vomiting, and hair loss); (c) grade 2 or above nonhematological toxicity for more than 3 weeks; (d) grade 4 neutropenia lasting at least 3 days, or neutropenia with fever ≥ 38.5°C; and (e) grade 4 thrombocytopenia or grade 3 thrombocytopenia with bleeding tendency. During the first 28 days of the administration period, if the DLT occurred in more than one third of the subjects in a dose group, the previous dose group was defined as the MTD in this trial. The clinical response after combination treatment was evaluated by computed tomography (CT) and was categorized as a complete response (CR), partial response (PR), stable disease (SD), or progressive disease (PD), according to the RECIST 1.1 criteria.

### 2.12 Statistical analysis

For in vitro studies, the differences between/among groups were analyzed using unpaired two‐tailed *t*‐tests, two‐way ANOVAs, or factorial analysis by GraphPad Prism version 7.0. For the clinical trial, all statistical analyses were performed with SAS 9.2 software. The difference between groups was analyzed using ANOVA or unpaired two‐tailed *t*‐tests. The *P*‐value no more than .05 was considered statistically significant. The confidence interval is 95%.

## 3 RESULTS

### 3.1 Pyrotinib inhibits cell proliferation in HER2‐positive GC cells via blocking the AKT/S6 pathway

In vitro analysis showed that HER2‐positive GC cells were more sensitive to pyrotinib than HER2‐negative GC cells (Figs. S1A and S1B). Moreover, compared with the positive control of lapatinib approved for HER2‐positive BC, the IC_50_ values of pyrotinib were lower in GC cell lines (Figs. S1A and S1B). We also found that pyrotinib might play its inhibitory effect via blocking the downstream AKT/S6 pathway indicated as decreased phosphorylated AKT and S6 rather than ERK in HER2‐positive NCI‐N87 cells at 10 nM (Fig. S1C).

### 3.2 Pyrotinib exerts powerful antitumor activity in HER2‐positive AVATAR mouse

**FIGURE 1:**
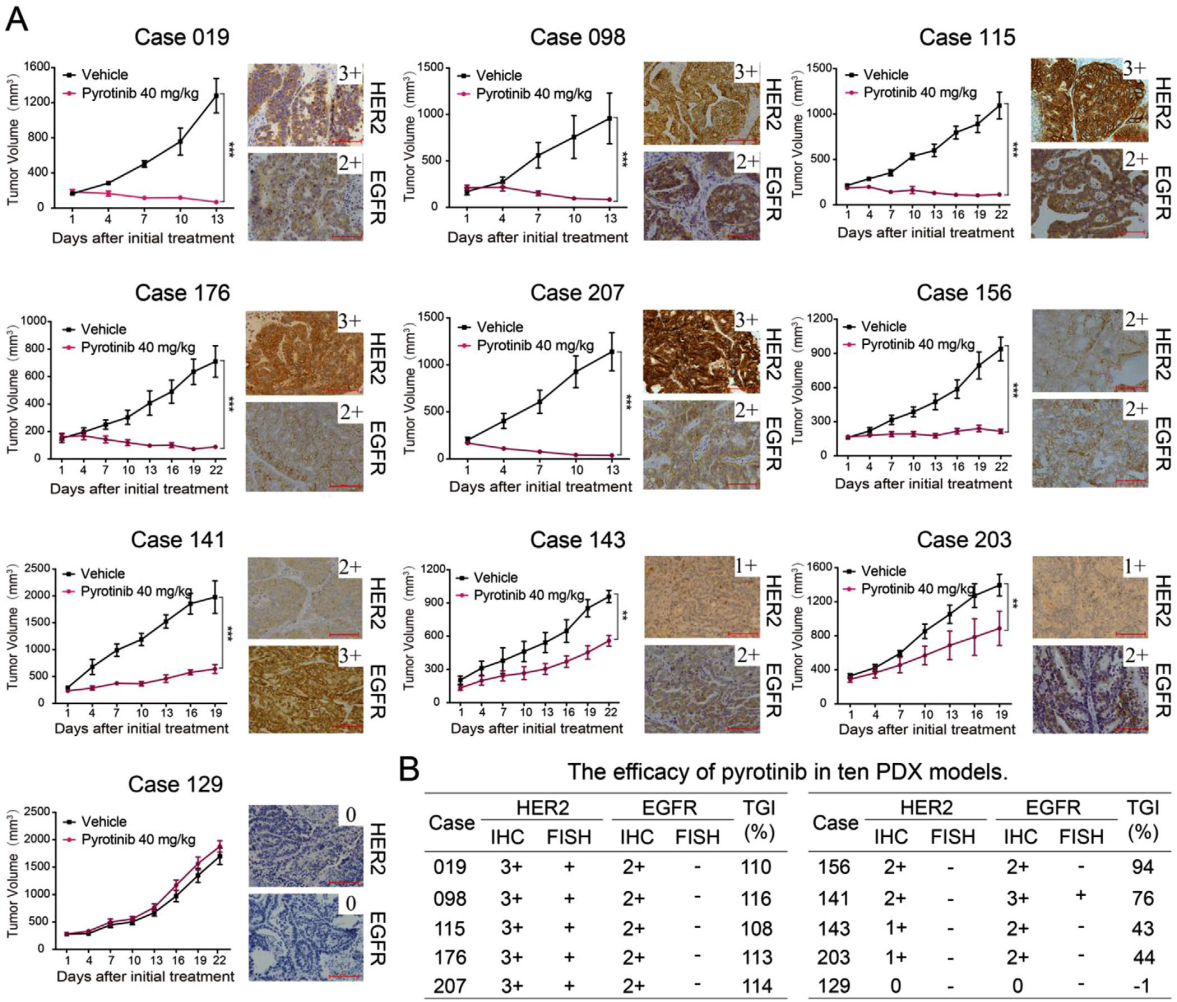
Pyrotinib exerts selective antitumor activity in GC AVATAR models. A, Antitumor activity of pyrotinib in 10 GC AVATAR models with different expression of HER2 and EGFR (×200 magnification; scale bar represents 100 µm). Data are presented as mean ± SEM (n = five mice per group). B, The efficacy of pyrotinib in 10 AVATAR models with different molecular features of HER2 and EGFR IHC, immunohistochemistry; FISH, fluorescence in situ hybridization; TGI, tumor growth inhibition.

AVATAR mice from 10 patients with different molecular features were exploited to evaluate the antitumor activity of pyrotinib in vivo. Compared with those of the vehicle groups, pyrotinib induced tumor regression in 5 HER2‐positive (IHC 3+ and FISH +) AVATAR models with high TGI ranging from 108% to 116%. Pyrotinib exerted a relatively strong antitumor effect in Case 156 (TGI = 94%) with moderate expressions of HER2 (IHC 2+/FISH–) and EGFR (IHC 2+/FISH–), and Case 141 (TGI = 76%) with low‐HER2 expression (IHC 1+/FISH–) and high‐EGFR expression (IHC 3+/FISH+). However, in the rest 3 AVATAR models with low expressions of HER2 and EGFR, very weak or no tumor inhibition were found (Figure 1A‐C).

### 3.3 Establishment and characteristics of the pyrotinib refractory AVATAR model

**FIGURE 2:**
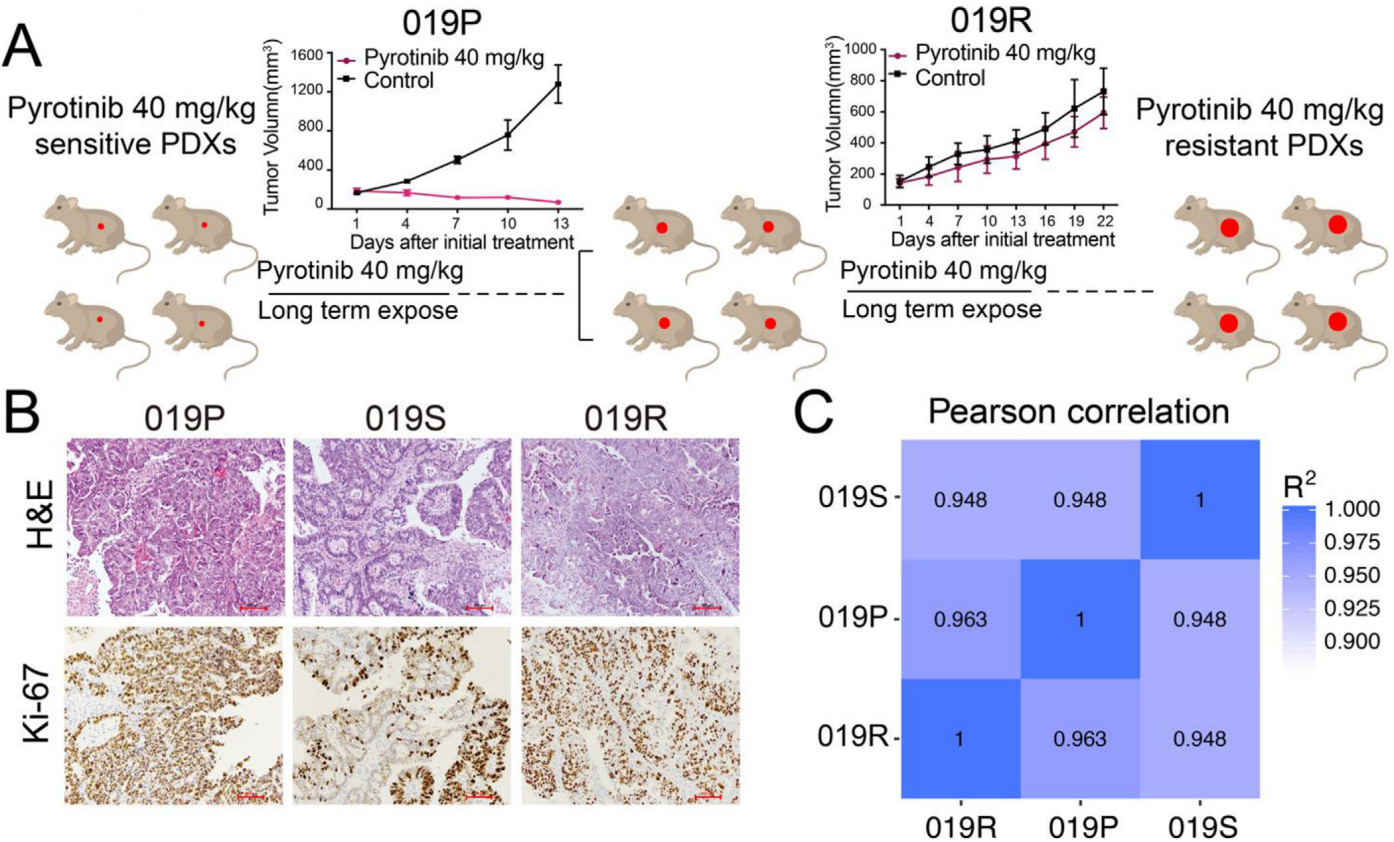
The establishment and characteristics of the pyrotinib‐refractory AVATAR model. A, The process of generating a pyrotinib‐refractory AVATAR model (019R). B, H&E staining and immunohistochemical staining for Ki‐67 in 019P, 019S, and 019R (×100 magnification; scale bar represents 100 µm). C, The Pearson correlation between 019P, 019S, and 019R by transcriptome sequencing

Due to the complexity of the patients, pyrotinib did not work well in clinical practice. We speculated that a pyrotinib‐combined strategy was needed to be explored in GC. One parental model Case 019 (named as 019P), which was sensitive to pyrotinib (Figures 1A and 1B), was chosen and initially treated with pyrotinib (40 mg/kg, daily by oral gavage) for 2 weeks followed by tumor isolation (named as 019S) and inoculation into new mice. When tumor volume reached 150‐250 mm^3^ again, mice were given pyrotinib 40 mg/kg for 4 weeks followed by another three passages until the tumor tissue was no longer sensitive to pyrotinib, indicating that the tumor was refractory to pyrotinib (named as 019R; Figure 2A). H&E staining showed consistent pathological morphology among tumor tissues from 019P, 019S, and 019R (Figure 2B). Ki‐67 staining indicated that compared to 019P, cell proliferation decreased in 019S tissues that shrunk quickly under pyrotinib treatment, but increased again in pyrotinib refractory 019R tissues (Figure 2B). Moreover, transcriptomic sequencing recapitulated the high homology between tissues before and after pyrotinib refractory, indicated by a high Pearson correlation coefficient (Figure 2C).

### 3.4 Dysregulation of the CCND1‐CDK4/6‐Rb axis contributes to pyrotinib resistance and CDK4/6 inhibitor SHR6390 sensitizes pyrotinib in AVATAR model

**FIGURE 3:**
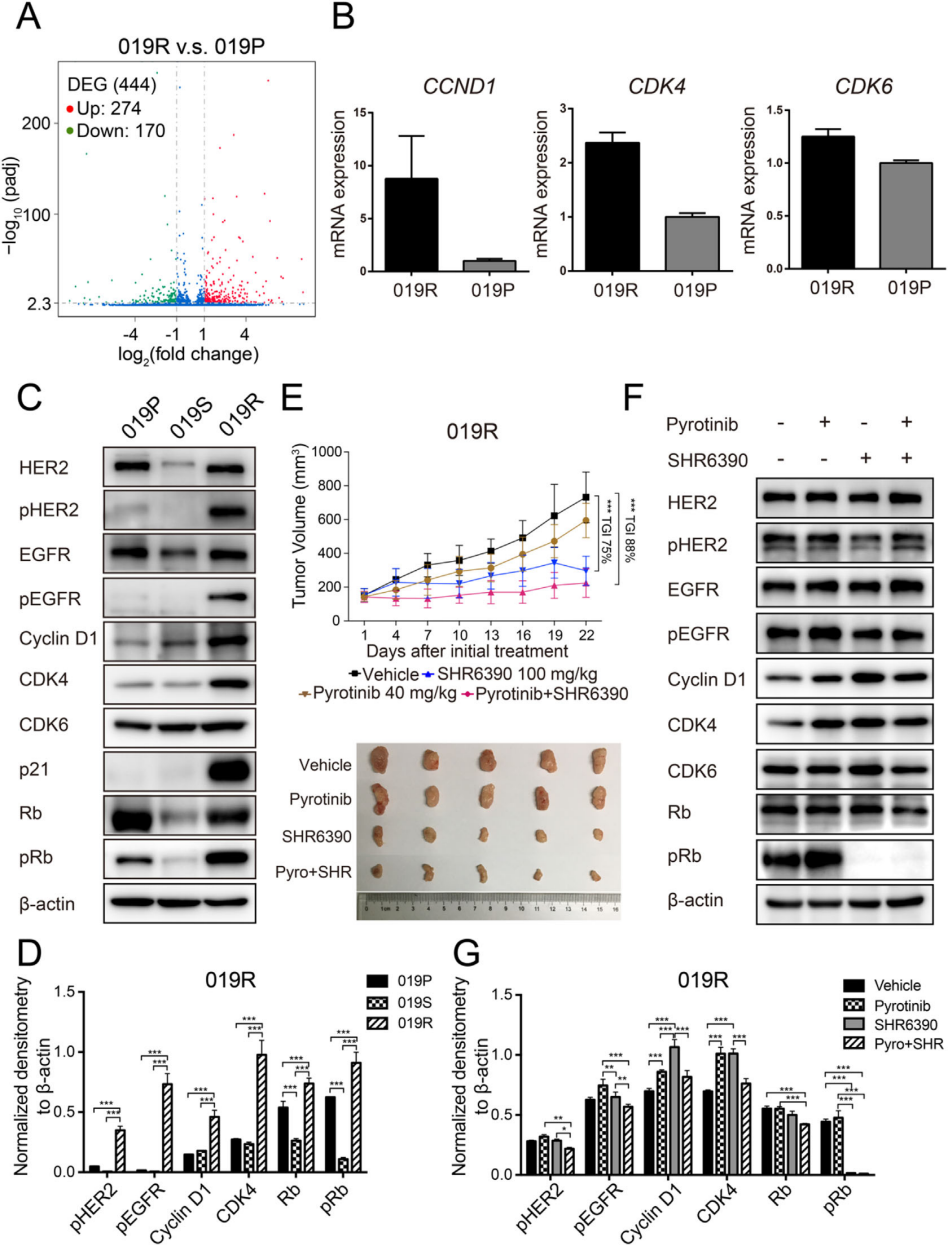
CDK4/6 inhibitor SHR6390 sensitizes pyrotinib in pyrotinib refractory AVATAR model. A, Differentially expressed genes (DEGs) in the 019S and 019R detected by RNA sequencing. Green, *P*_adj_ < .005 and log_2_ (fold change) < –1; Red, *P*_adj_ < .005 and log_2_ (fold change) > 1. B, Quantitative real‐time PCR of *CCND1*, *CDK4*, and *CDK6* in 019P and 019R. Data are presented as mean ± SD of three independent experiments. C and D, Expression and quantification of critical molecules in the ErbB family and cell‐cycle signaling pathway in 019P, 019S, and 019R. E, The efficacy of pyrotinib, SHR6390, and the combination therapy in 019R. Data are presented as mean ± SD (n = 5 mice per group). F and G, Expression and quantification of critical molecules in the ErbB family and cell cycle signaling pathway in 019R. *P < .05, **P < .01, and ***P < .001 according to repeated measures ANOVAs

No obvious variations were found in tumor tissues between 019P and 019R by next‐generation DNA sequencing of 483 genes (Fig. S2). However, compared with those of 019P, tumor tissues of 019R showed distinct gene expression profiles that included 274 upregulated and 170 downregulated genes based on RNA sequencing (Figure 3A). The expressions of *CCND1* and *CDK4* were significantly upregulated in 019R tissue compared to 019P tissue in mRNA level (Figure 3B), which was further confirmed in protein level (Figure 3C). Even more, the phosphorylated Rb, a direct marker of CDK4/6 activity, was also increased in 019R tissue, which verified the evidence of activated CCND1‐CDK4/6‐Rb axis involved in pyrotinib resistance (Figures 3C and 3D). Based on above results, the antitumor activity of SHR6390 (a CDK4/6 inhibitor) alone or in combination with pyrotinib was evaluated in pyrotinib refractory 019R AVATAR mouse. Compared SHR6390 alone (TGI = 75%; Figure 3E), SHR6390 combined with pyrotinib showed the best antitumor activity (TGI = 88%). In addition, the immunoblot result showed that SHR6390 alone or in combination with pyrotinib induced the significant suppression of Rb phosphorylation (Figures 3F and 3G).

### 3.5 Pyrotinib combined with SHR6390 showed a promising response rate in HER2‐positive AGC patients

A phase I clinical trial (NCT03480256) was conducted and five GC patients who failed with systematic treatments were enrolled to receive pyrotinib combined with SHR6390. The characteristics of these GC patients were shown in Table 1 and all of them were initially treated with 100 mg/day of SHR6390 and 400 mg/day of pyrotinib.

**TABLE 1:** The clinical characteristics of the enrolled AGC patients

ID	Gender	Age	TNM	Primary Site	Metastatic Site	Differentiation	Lauren classification	HER2 IHC	EGFR IHC	Prior therapies
1	Female	60	pT4N1 M0→M1	Cardia	Liver Lung Bone	Low to Medium	Mixed	3+	1+	First: oxaliplatin + leucovorin calcium + tegafur Second: paclitaxel + loplatin Third: leucovorin calcium + 5‐fluorouracil + irinotecan + apatinib Fourth: docetaxel + capecitabine Fifth: paclitaxel liposomes
2	Female	41	pT3N3b M0→M1	Antrum	Axillary LN Supraclavicular LN	Low to Medium	Diffused	3+	1+	First: oxaliplatin + capecitabine Second: RC48‐ADC Third: irinotecan + trastuzumab
3	Male	59	pT4aN3aM0→M1	Cardia	Pleura Lung	Low to Medium	Intestinal	3+	3+	First line: S‐1 Second: paclitaxel + capecitabine
4	Male	35	cTxN+M1	Antrum	Liver Left adrenal gland Multiple LNs	Medium	Intestinal	3+	3+	First: docetaxel + oxaliplatin + apatinib Second: paclitaxel + oxaliplatin + capecitabine + trastuzumab
5	Male	65	pT2N2 M0→M1	Antrum	Liver Multiple LNs	Low to Medium	Intestinal	3+	NA	First: oxaliplatin + S‐1 Second: paclitaxel + capecitabine + trastuzumab

LN, lymph nodes; T, size or direct extent of the primary tumor; N, degree of spread to regional lymph nodes; M, presence of distant metastasis.

**FIGURE 4:**
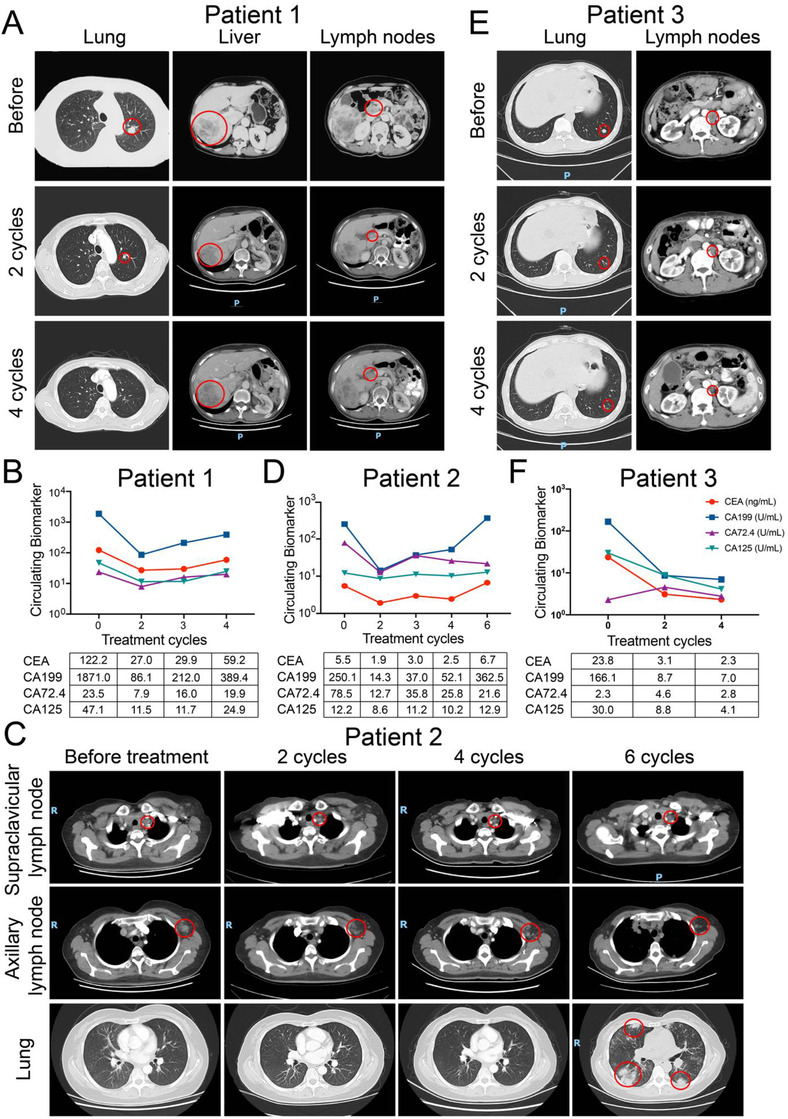
The clinical responses of three HER2‐positive GC patients treated by pyrotinib combined with SHR6390. A, CT scans of tumor metastases in lung, liver, and hepatic hilar lymph nodes during four cycles of combination treatment in patient 1. C, CT scans of tumor metastases in lung, axillary, and supraclavicular lymph nodes during six cycles of combination treatment in patient 2. E, CT scans of tumor metastases in lung and retroperitoneal lymph nodes during four cycles of combination treatment in patient 3. B, D, and F, The dynamic change of CEA, CA199, CA72.4, and CA125 during combination treatment in patient 1, 2, and 3

**FIGURE 5:**
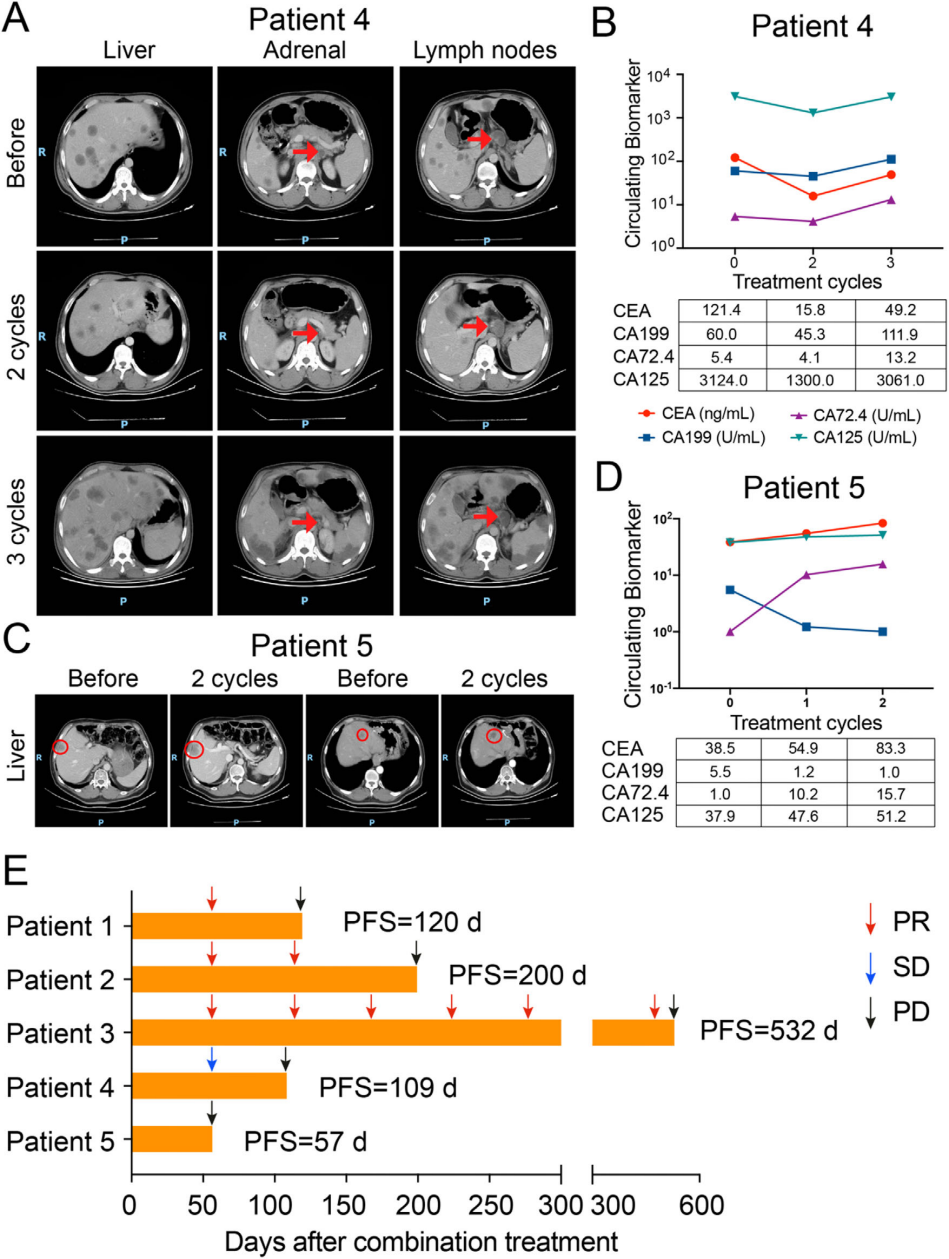
The clinical responses of the other two HER2‐positive GC patients treated by pyrotinib combined with SHR6390. A, CT scans of tumor metastases in liver, left adrenal gland, and perigastric lymph nodes during three cycles of combination treatment in patient 4. C, CT scans of tumor metastases in liver during two cycles of combination treatment in patient 5. B and D, The dynamic change of CEA, CA199, CA72.4, and CA125 during combination treatment in patient 4 and 5. E, Treatment response of each patient after combination treatment and the duration of response. Patient number are shown to the left of the *Y*‐axis PR, partial response; SD, stable disease; PD, progressive disease. PFS, progression‐free survival.

Until the last follow‐up of June 2020, the best response was PR in three patients, SD in one patient, and PD in one patient. The progression‐free survival (PFS) time was 120, 200, 532, 109, and 57 days, respectively. All these five patients had previously undergone multiple systematic treatments of chemotherapy or anti‐HER2 therapy. After two cycles of pyrotinib plus SHR6390 treatment, multiple target lesions in patient 1, 2, and 3 significantly reduced in size accompanied by decreased tumor biomarkers with the clinical response of PR (Figure 4A‐F). Patient 4 achieved a clinical response of SD and developed grade 4 neutropenia. Then he received pyrotinib of 320 mg/day (Figures 5A and 5B). Patient 5 progressed quickly with increased liver metastasis after 2 cycles of treatment (Figures 5C and 5D).

As shown in Figure 5E, for patient 1, the disease progressed after a total of four cycles of treatment, with the PFS 120 days. For patient 2, after six cycles of treatment, the lung metastases were newly developed and the PFS was 200 days. But for patient 3, the disease progressed after 19 cycles of treatment and the PFS was 532 days. For patient 4, the disease progressed after three cycles of treatment with the PFS of 109 days. For patient 5 who was primarily resistant to treatment, the PFS was 57 days. Among these five patients, the hematological adverse events were leucopenia (grade 2 to 3), neutropenia (grade 1 to 4), anemia (grade 1 to 3), and thrombocytopenia (grade 1). We observed DLT only in patient 4, with a grade 4 of neutropenia. The nonhematological adverse events included oral ulcer, fatigue, rash, interstitial pneumonia, and nausea, which were judged as grade 1 to 2.

## 4 DISCUSSION

The ErbB family is one of the most common targeted families due to its aberrant activation in numerous cancers. After the breakthrough of trastuzumab,^12^ other compounds targeting ErbB members including pertuzumab,^13^, ^14^ trastuzumab emtansine,^15^ and pyrotinib^8^ have been confirmed to be effective in HER2‐positive patients with metastatic BC. However, the high heterogeneity hinders the development of anti‐HER2 treatments for GC,^16^, ^17^ which remains as a focus for novel drug developments. Preclinical studies have demonstrated that, compared to single‐target drugs, pan‐HER blockade induced sustained inhibition of HER3 and EGFR^18^ and could overcome intrinsic or acquired resistance,^19^ which suggested the prospective use of this strategy in clinical practice.

Pyrotinib is a Pan‐HER tyrosine kinase inhibitor that irreversibly blocks EGFR, HER2, and HER4. We found that pyrotinib significantly inhibited cell proliferation in HER2‐positive GC cell lines and tumor growth in AVATAR models via inactivating the downstream AKT/S6 pathway. However, based on the data of clinical trial NCT02378389, the efficacy of pyrotinib in HER2‐positive GC patients was unsatisfactory, although it was well tolerated. Identification of an optimal preclinical model was necessary for translational studies. The pyrotinib‐refractory AVATAR model had been consequently established by long‐term intermittent exposure of pyrotinib. By high‐throughput analysis, the aberrantly activated CCND1‐CDK4/6‐Rb axis was selected and validated to be involved in pyrotinib‐refractory GC AVATAR model. Furthermore, we confirm that the CDK4/6 inhibitor SHR6390 significantly sensitized pyrotinib in the pyrotinib‐refractory AVATAR model, which was similar to the result in the study of HER2‐positive BC.^20^

SHR6390 is a small‐molecular, oral potent, selective CDK4/6 inhibitor. A one‐arm, open, dose escalation phase I study (NCT02684266) to investigate the safety and the pharmacokinetic profile of SHR6390 has been conducted in patients with advanced solid tumors. Nineteen patients were enrolled. Five cycles of treatment were received on average and the DLT of SHR6390 was not observed even in the 175 mg/day dose level. Given the fact that pyrotinib and SHR6390 were safe and tolerable in patients, we conducted the phase I clinical trial (NCT03480256) to validate the safety and efficacy of pyrotinib combined with SHR6390 in GC patients.

Up to now, five patients were enrolled and had available response evaluation after treated by pyrotinib plus SHR6390. After two cycles of treatment, three patients achieved PR, one patient achieved SD, and one patient achieved PD. Also, any adverse reactions were tolerated. Although only five patients have been treated, our results suggested that the strategy of CDK4/6 inhibitor combined with anti‐HER2 agents was promising for GC treatment in the future. Up to date, two CDK4/6 inhibitors, palbociclib and abemaciclib, have been approved for treatment of BC.^21^ Recently, the clinical investigations of CDK4/6 inhibition and HER2 blockade have been explored in HER2‐positive BC.^22^ The combination of CDK4/6 inhibitor and anti‐HER2 treatment showed high response rate and was well tolerated in BC patients,^23^, ^24^ which was consistent with the results of our present study in GC patients. In addition, cyclin D1‐CDK4/6 pathway may be a potential therapeutic target for patients with resistant nonsmall cell lung cancer (NSCLC). It has been reported that transcriptional activation of cyclin D1 via HER2/HER3 contributed to EGFR‐TKI resistance in lung cancer cells.^25^ A phase 1/2 study (NCT03455829) has been conducted to investigate the potential clinical benefit of G1T38 combined with osimertinib in patients with EGFR mutation‐positive metastatic NSCLC.

The CCND1‐CDK4/6‐Rb axis was well‐known to be implicated in all cancers and played its role through cascade reactions.^26^ In our exploration of mechanisms, SHR6390 treatment reduced the phosphorylation of Rb, which was a direct marker of CDK4/6 activity. This result was consistent with results from other studies in BC.^20^ Besides aberrant cell cycle, other refractory mechanisms including bypassing activation,^27^, ^28^ activation of PIK3CA mutation,^29^ and expression of Mucin 4^30^ had also been reported. In this study, upregulations of HER3 and MET and reactivation of AKT/S6 and MAPK signaling pathways were detected in pyrotinib refractory tissues compared to those in pyrotinib sensitive tissues (Fig. S3), which was also consistent with previous literature.^18^ Due to the complication of the pyrotinib‐refractory mechanisms, our results also suggested multiple combinations of therapeutic strategy, which deserved further investigation.

## ETHICS APPROVAL AND CONSENT TO PARTICIPATE

This study was approved by the Medical Ethics Committee of Peking University Cancer Hospital. All animal studies complied with the ARRIVE guidelines and were conducted in accordance with the UK Animals (Scientific Procedures) Act, 1986 and associated guidelines, EU Directive 2010/63/EU for animal experiments, or the National Institutes of Health guide for the care and use of Laboratory animals (NIH Publications No. 8023, revised 1978). Experiments involving humans were in accordance with the ethical standards of committees (institutional and national) and with The Code of Ethics of the World Medical Association (Declaration of Helsinki). All patients completed written informed consent prior to study entry.

5

## CONFLICT OF INTERESTS

Jianjun Zou and Xiaoyu Zhu are employees from Jiangsu Hengrui Medicine Co, Ltd. All other authors have no conflict of interest.

## AUTHOR CONTRIBUTIONS

Lin Shen, Jing Gao, Jianjun Zou, and Xiaoyu Zhu conceived and designed the study. Zuhua Chen, Yingying Xu, Jifang Gong, Furong Kou, and Tiantian Tian performed the clinical trials and in vitro experiments. Mengqi Zhang and Cheng Zhang contributed reagents and materials. Zhongwu Li and Yumei Lai contributed to data analysis. Jifang Gong, Xiaotian Zhang, and Jian Li conducted the clinical study. Zuhua Chen, Jing Gao, and Lin Shen wrote and revised the manuscript. All of the authors read and approved the final manuscript.

## Supporting information

Figure S1

Figure S2

Figure S3

Figure Caption

Table S1–S4

## Data Availability

Most data relevant to the study are included in the article or uploaded as Supporting Information. Others are available on request from the corresponding author.
