# Supplementary figures and images for: Pyrotinib combined with CDK4/6 inhibitor in HER2‐positive metastatic gastric cancer: A promising strategy from AVATAR mouse to patients
Source: Clin Transl Med. 2020 Aug 13;10(4):e148. doi: 10.1002/ctm2.148 (PMC7424666; doi:10.1002/ctm2.148)

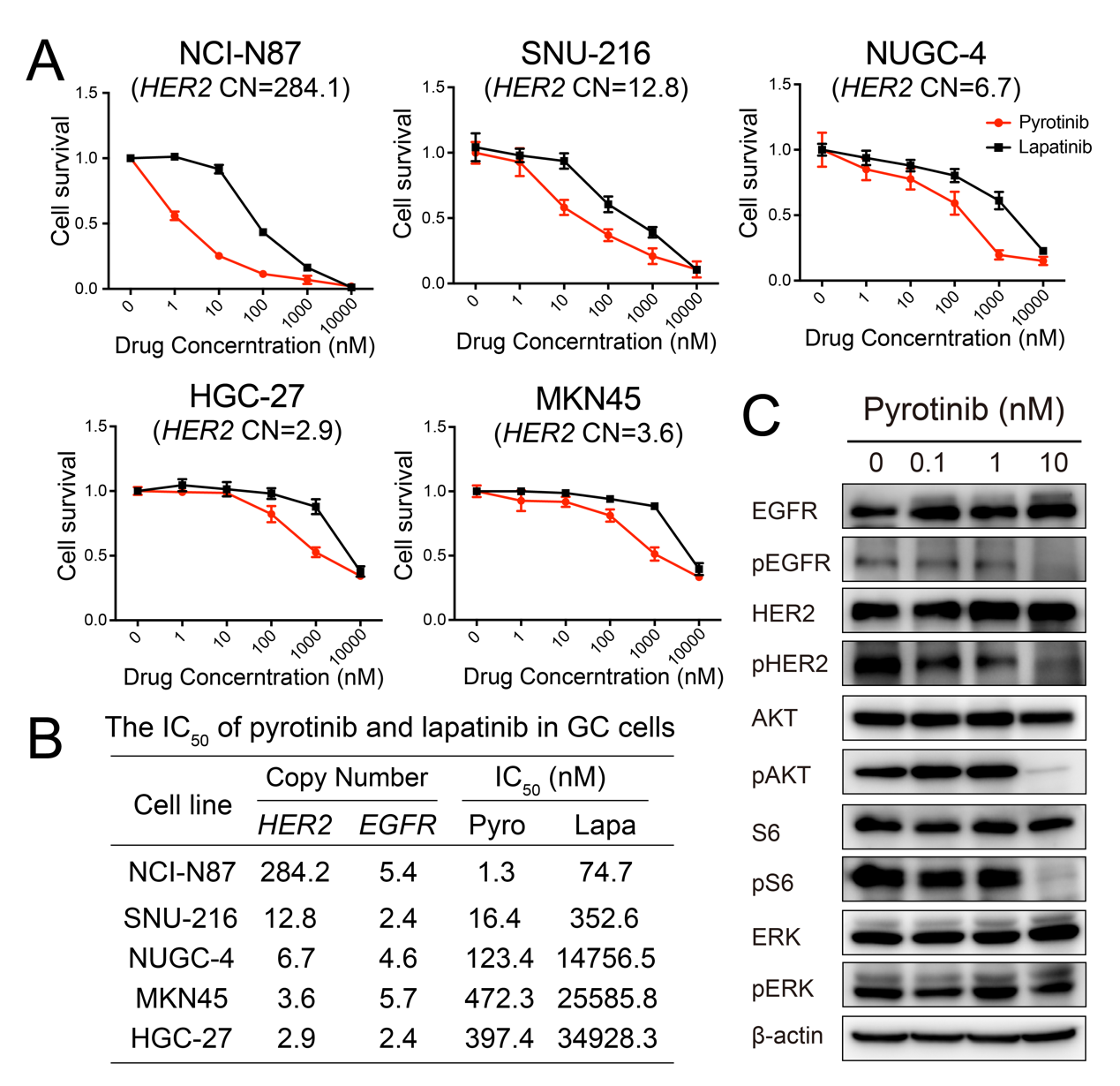

Supplement: Supplementary file 1 — Figure S1 [file CTM2-10-e148-s001.docx]

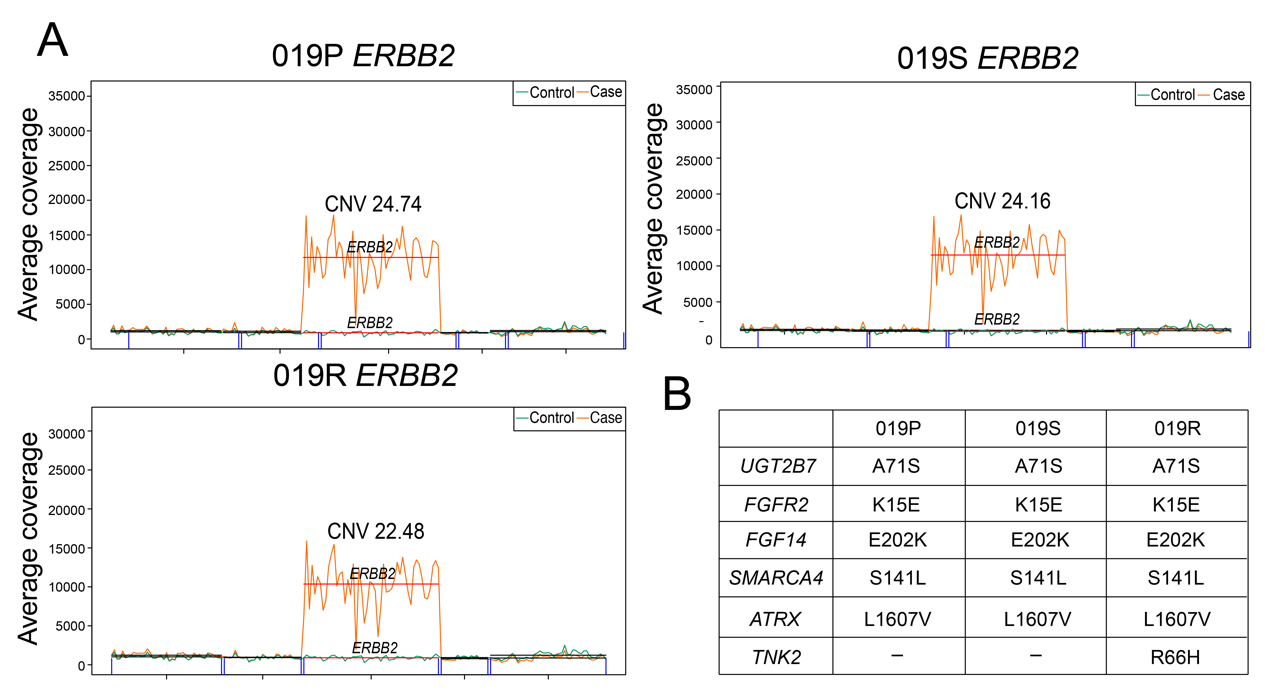

Supplement: Supplementary file 2 — Figure S2 [file CTM2-10-e148-s002.docx]

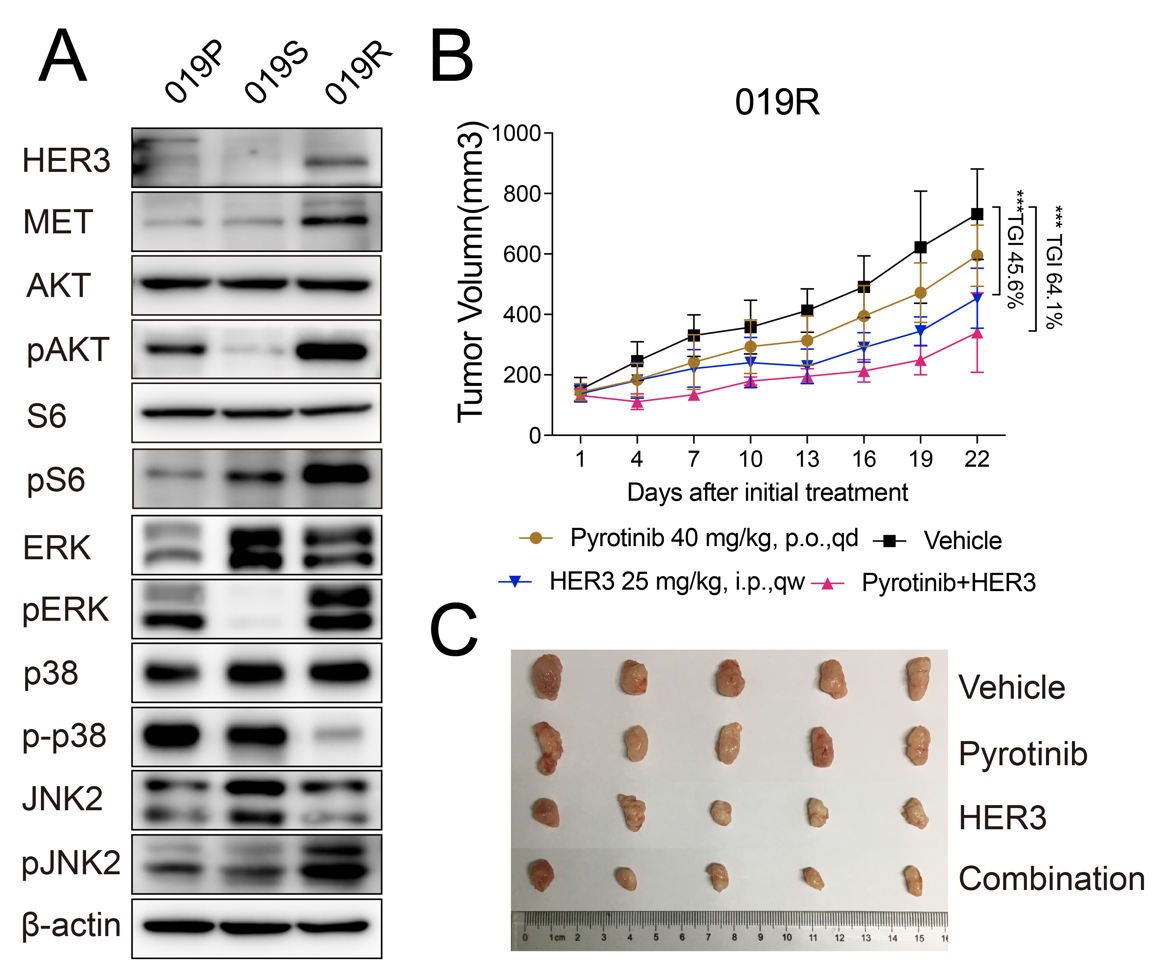

Supplement: Supplementary file 3 — Figure S3 [file CTM2-10-e148-s003.docx]
