# Supplementary material for: Pyrotinib combined with CDK4/6 inhibitor in HER2‐positive metastatic gastric cancer: A promising strategy from AVATAR mouse to patients
Source: Clin Transl Med. 2020 Aug 13;10(4):e148. doi: 10.1002/ctm2.148 (PMC7424666; doi:10.1002/ctm2.148)
Supplement: Supplementary file 4 — Figure Caption [file CTM2-10-e148-s004.docx]

**Pyrotinib combined with CDK4/6 inhibitor in HER2-positive metastatic gastric cancer: a promising strategy from AVATAR mouse to patients**

Legend for Supplementary Figures:

**Fig. S1 Pyrotinib significantly inhibits cell proliferation in HER2-positive GC cells via blocking the AKT/S6 pathway.** **(A)** Cell viability of five GC cell lines treated with pyrotinib and lapatinib for 72 h. Data are presented as means ± SDs of three independent experiments. **(B)** The IC50 values of pyrotinib (Pyro) and lapatinib (Lapa) in GC cells with different copy numbers of HER2 and EGFR. **(C)** Phosphorylation of EGFR, HER2, AKT, S6 and ERK, detected by immunoblotting after pyrotinib treatment for 24 h in NCI-N87 cells.

**Fig. S2 Next-generation sequencing results of tumor tissues derived from 019P, 019S and 019R.** **(A)** The copy number variation of ERBB2 in parental, pyrotinib-sensitive, and pyrotinib-resistant models. CNV, copy number variation. **(B)** The non-synonymous single-nucleotide variations in parental, pyrotinib-sensitive, and pyrotinib-resistant models.

**Fig. S3 Other potential mechanisms and therapeutic therapies in the pyrotinb refractory model.** **(A)** The upregulation of HER3 and MET, and activation of AKT/S6 and MAPK signaling pathways in the pyrotinb refractory model. **(B)** The antitumor activity of HER3 blockade combined with pyrotinib in the pyrotinb refractory model.
