# Supplementary material for: Pyrotinib combined with CDK4/6 inhibitor in HER2‐positive metastatic gastric cancer: A promising strategy from AVATAR mouse to patients
Source: Clin Transl Med. 2020 Aug 13;10(4):e148. doi: 10.1002/ctm2.148 (PMC7424666; doi:10.1002/ctm2.148)
Supplement: Supplementary file 5 — Table S1–S4 [file CTM2-10-e148-s005.docx]

**Supplementary materials**

**The safety and efficacy of pyrotinib** **in HER2-positive gastric cancer in phase I clinical trial of NCT02378389**

**Overview of study design:** A traditional 3+3 design was used for dose escalation of pyrotinib. Pyrotinib was administered orally once daily in 21-day cycles. Planed dose escalation was 240, 320, 400, and 480 mg. Patients with advanced gastric cancer after failure of systematic treatment or intolerable of prior treatments were eligible for enrollment as following: 18-70 years of age, pathological confirmed HER2 positive (immunochemistry 3+, or immunochemistry 2+ confirmed positive by fluorescent in situ hybridization), eastern cooperative oncology group scale of 0 to 1, life expectancy of more than 12 weeks, at least one measurable lesion [response evaluation criteria in solid tumors (RECIST) version 1.1], adequate bone marrow and organ function.

The DLT (dose-limiting toxicity) evaluation started from the first administration of pyrotinib and continued until day 21 (within the first cycle). DLTs were defined as any of the following AEs (adverse events) related to treatment: grade 4 leukopenia or neutropenia with a duration of ≥7 days; grade 3 or 4 neutropenia with fever ≥38.5°C; grade 3 thrombocytopenia with bleeding tendency or grade 4 thrombocytopenia; grade 3 or 4 anemia; grade 2 heart failure; grade 2 renal failure; or grade 3 or 4 other non-hematologic AEs. Safety of pyrotinib was assessed by evaluation of AEs using national cancer institute common terminology criteria for adverse events (CTCAE) version 4.0. Efficacy evaluation was performed in accordance with RECIST version 1.1 every two cycles. Complete response (CR) or partial response (PR) had to be confirmed at least 4 weeks after initial response.

**Main findings:** Between September 2014 and February 2017, 15 patients received pyrotinib therapy with 3 patients in 240mg group, 3 patients in 320mg group, 6 patients in 400mg group, and 3 patients in 480 mg group. DLT was only found in one patient with grade 3 diarrhea at 400 mg group. All patients had treatment-related adverse events (TRAEs), and the most frequently TRAEs were diarrhea (12/15, 80%) and anemia (5/15, 33%). Most AEs were grade 2 or lower, only four (27%) patients had grade ≥3 TRAEs of diarrhea (2/15, 13%), neutropenia (1/15, 7%), increased transaminase (1/15, 7%) or increased bilirubin (1/15, 7%) (**Table S1**).

Finally, fourteen patients were evaluable for efficacy, and there were 3 patients and 3 patients who had confirmed partial response (PR) and stable disease (SD), respectively, with the overall response rate (ORR) of 21% (3/14) and disease control rate (DCR) of 43% (6/14, **Table S2**). Although the ORR and DCR were not very satisfied, there was one patient who benefited from pyrotinib with PFS (progression-free survival) of 614 days (**Table S3**).

**Table S1. Treatment-related adverse events of all grades**

| **Adverse Event** | **240 mg, n=3** | | **320 mg, n=3** | | **400 mg, n=6** | | **480 mg, n=3** | | **Total, n=15** | |
| --- | --- | --- | --- | --- | --- | --- | --- | --- | --- | --- |
|  | **All Grade**  **n (%)** | **G3-4**  **n (%)** | **All Grade**  **n (%)** | **G3-4**  **n (%)** | **All Grade**  **n (%)** | **G3-4**  **n (%)** | **All Grade**  **n (%)** | **G3-4**  **n (%)** | **All Grade**  **n (%)** | **G3-4**  **n (%)** |
| Hematologic toxicity |  |  |  |  |  |  |  |  |  |  |
| Leukopenia |  |  | 2(67) |  | 2(33) |  |  |  | 4(27) |  |
| Neutropenia | 1(33) |  | 2(67) |  | 1(17) | 1(17) |  |  | 4(27) | 1(7) |
| Anemia | 1(33) |  | 1(33) |  | 2(33) |  | 1(33) |  | 5(33) |  |
| Thrombocytopenia |  |  |  |  | 1(17) |  |  |  | 1(7) |  |
| Non-hematologic toxicity |  |  |  |  |  |  |  |  |  |  |
| Diarrhea | 2(67) |  | 3(100) |  | 5(83) | 2(33) | 2(67) |  | 12(80) | 2(13) |
| Increased bilirubin | 2(67) |  | 1(33) | 1(33) |  |  |  |  | 3(20) | 1(7) |
| Fatigue | 1(33) |  | 1(33) |  | 1(17) |  |  |  | 3(20) |  |
| Nausea | 1(33) |  |  |  | 2(33) |  |  |  | 3(20) |  |
| Anorexia | 1(33) |  |  |  | 2(33) |  |  |  | 3(20) |  |
| Acid regurgitation | 1(33) |  | 1(33) |  | 1(17) |  |  |  | 3(20) |  |
| Abdominal discomfort |  |  | 1(33) |  | 2(33) |  |  |  | 3(20) |  |
| Abdominal pain |  |  |  |  | 2(33) |  |  |  | 2(13) |  |
| Rash |  |  | 1(33) |  |  |  | 1(33) |  | 2(13) |  |
| Heartburn |  |  | 1(33) |  | 1(17) |  |  |  | 2(13) |  |
| Increased transaminase |  |  | 1(33) | 1(33) |  |  |  |  | 1(7) | 1(7) |
| Increased creatinine |  |  |  |  | 1(17) |  |  |  | 1(7) |  |
| Cutaneous pruritus |  |  |  |  | 1(17) |  |  |  | 1(7) |  |
| Weight loss |  |  |  |  | 1(17) |  |  |  | 1(7) |  |
| Hematochezia |  |  | 1(33) |  |  |  |  |  | 1(7) |  |
| Fullness in head | 1(33) |  |  |  |  |  |  |  | 1(7) |  |
| Dysgeusia |  |  |  |  | 1(17) |  |  |  | 1(7) |  |

**Table S2. Overall response in the evaluable population**

| **Dose cohort** | **Efficacy** | | | | | |  |
| --- | --- | --- | --- | --- | --- | --- | --- |
|  | **CR** | **PR** | **SD** | **PD** | **ORR, n (%)** | **DCR, n (%)** | |
| 240 mg (n=3) | 0 | 1 | 0 | 2 | 1(33) | 1(33) | |
| 320 mg (n=3) | 0 | 0 | 1 | 2 | 0(0) | 1(33) | |
| 400 mg (n=5^a^) | 0 | 1 | 1 | 3 | 1(20) | 2(40) | |
| 480 mg (n=3) | 0 | 1 | 1 | 1 | 1(33) | 2(67) | |
| Total (n=14) | 0 | 3 | 3 | 8 | 3(21) | 6(43) | |

Abbreviations: CR, complete response; PR, partial response; SD, stable disease; PD, progressive disease; ORR, overall response rate: (CR+PR)/Total; DCR, disease control rate: (CR+PR+SD)/Total. ^a^Six patients were enrolled 400 mg dose cohort. One patient was not evaluable.

**Table S3. Progression-free survival of patients who had response of PR or SD**

| **Dose Cohort** | **Best ORR** | **Progression-Free Survival (days)** | **Prior Trastuzumab Therapy** |
| --- | --- | --- | --- |
| 240 mg | PR | 125 | No |
| 320 mg | SD | 81 | Yes |
| 400 mg | PR | 614 | No |
| 400 mg | SD | 128 | Yes |
| 480 mg | PR | 171 | No |
| 480 mg | SD | 80 | No |

Abbreviations: ORR: overall response rate; PR, partial response; SD, stable disease.

**Table S4. Sequences of PCR primers.**

| Genes | Primers | Length |
| --- | --- | --- |
| CCND1 | Forward: 5’-AACACGGCTCACGCTTAC-3’ | 201bp |
|  | Reverse: 5’-CCAGACCCTCAGACTTGC-3’ |  |
| CDK4 | Forward: 5’-AATGTTGTACGGCTGATGGA-3’ | 167bp |
|  | Reverse: 5’-AGAAACTGACGCATTAGATCCT-3’ |  |
| CDK6 | Forward: 5’-CCAGATGGCTCTAACCTCAGT-3’ | 124bp |
|  | Reverse: 5’-AACTTCCACGAAAAAGAGGCTT-3’ |  |
| GAPDH | Forward: 5’-AGGGCTGCTTTTAACTCTGGT-3’ | 206bp |
|  | Reverse: 5’-CCCCACTTGATTTTGGAGGGA-3’ |  |
